# Trends in electroconvulsive therapy and repetitive transcranial magnetic stimulation use in affective disorders in Germany: Evidence from national hospital remuneration data

**DOI:** 10.1016/j.nsa.2026.107032

**Published:** 2026-10-28

**Authors:** Kurt Noack, Marius Gruber, Marc Pawlitzki, Lars Masanneck, Christine Reif-Leonhard, Andreas Reif, Nils Opel, David Zilles-Wegner, Benjamin Friedrichson, Jonathan Repple

**Affiliations:** a Goethe University Frankfurt, University Hospital, Department of Psychiatry, Psychosomatic Medicine and Psychotherapy, Heinrich-Hoffmann-Straße 10, Frankfurt, 60528, Germany; b Goethe University Frankfurt, Cooperative Brain Imaging Center - CoBIC, Heinrich-Hoffmann-Straße 9, Frankfurt, 60528, Germany; c Institute for Translational Psychiatry, University of Münster, Robert-Koch-Straße 41/51, Münster, 48149, Germany; d Department of Neurology, Medical Faculty, University Hospital Düsseldorf, Heinrich Heine University Düsseldorf, Moorenstraße 5, Düsseldorf, 40225, Germany; e Fraunhofer Institute for Translational Medicine and Pharmacology ITMP, Theodor-Stern-Kai 7, Frankfurt, 60596, Germany; f Goethe University Frankfurt, Department of Anaesthesiology, Intensive Care Medicine and Pain Therapy, University Hospital Frankfurt, Theodor-Stern-Kai 7, 60590, Germany; g Goethe University Frankfurt, Institute of Medical Informatics (IMI), University Hospital Frankfurt, Theodor-Stern-Kai 7, Frankfurt, 60590, Germany; h Department of Psychiatry and Neurosciences, Campus Benjamin Franklin, Charité Universitätsmedizin Berlin, Bonhoefferweg 3, Berlin, 10117, Germany; i German Centre for Mental Health (DZPG), Site Berlin-Potsdam, Invalidenstraße 80, Berlin, 10115, Germany; j Department of Psychiatry and Psychotherapy, University Medical Center Göttingen, Von-Siebold-Straße 5, Göttingen, 37075, Germany

**Keywords:** ECT, rTMS, Unipolar depression, Bipolar depression, Affective disorder

## Abstract

Electroconvulsive therapy (ECT) remains the most effective acute treatment for severe forms of depression, including psychotic depression. Yet, its utilization is constrained and varies heavily across countries and years, especially given the increasing popularity of repetitive transcranial magnetic stimulation (rTMS). Therefore, this study analyzed nationwide German patient data (2020–2025) to assess temporal trends in ECT treatments relative to rTMS, indication-specific use, and regional variation of ECT.

We conducted a retrospective analysis of anonymized hospital remuneration data in adults (≥18 years) with unipolar or bipolar depression. Annual cases treated with ECT and rTMS were evaluated, alongside the proportion of patients with psychotic depression receiving ECT and state-level utilization rates.

Both modalities showed increases over time; however, growth differed substantially. rTMS exhibited a mean annual increase of 35.39% compared to 4.88% for ECT. Despite guideline recommendations, only few patients with psychotic depression received ECT (<6%), with small variation across years. ECT growth was primarily driven by recurrent severe non-psychotic depression. Marked regional disparities were observed, with ECT cases ranging from 2.45 to 7.26 cases per 100.000 inhabitants—representing almost a threefold difference across the German states (except for city states).

In conclusion, while utilization of both ECT and rTMS is rising, ECT remains underused in recurrent severe psychotic depression and unevenly distributed regionally. These findings highlight gaps in guideline adherence and suggest a need to expand ECT capacity and standardize access within the German healthcare system.

## 1 Introduction

Electroconvulsive Therapy (ECT) remains the benchmark for acute treatment of severe depression (Zilles-Wegner et al., 2024; Espinoza and Kellner, 2022), delivering robust response and remission rates across decades of research and clinical experience, yet its application is still underutilized (Thirthalli, 2025) and limited by stigma (Zilles-Wegner et al., 2026), anesthesia requirements, and the need for specialized inpatient or outpatient resources (Micallef-Trigona, 2014; Lauber et al., 2005). In contrast, repetitive Transcranial Magnetic Stimulation (rTMS) has gained prominence over the past two decades as a non-invasive, well-tolerated option—with primarily transient scalp discomfort and headache—supported by regulatory approvals, randomized controlled trials, and meta-analyses demonstrating superiority over sham stimulation and meaningful antidepressant effects (Micallef-Trigona, 2014; Adu et al., 2022; Nationale Versorgungs Leitlinie Unipolare Depression, n.d.). Head-to-head trials and syntheses indicate that ECT yields higher response and remission rates than high-frequency rTMS, particularly in severe or psychotic depression, while rTMS offers substantial efficacy in less refractory cases, superior cognitive outcomes, and greater patient preference (Micallef-Trigona, 2014; Timäus et al., 2021; Petrides et al., 2001; Strandberg et al., 2024). These attributes, alongside evidence of rTMS's neurophysiological and network-level effects, have spurred its earlier integration into treatment algorithms and the pursuit of personalized protocols (Nationale Versorgungs Leitlinie Unipolare Depression, n.d.; Klomjai et al., 2015). Meanwhile, shifts in healthcare delivery—including resource allocation and the COVID-19 pandemic—may have reshaped the availability and utilization of both modalities, though systematic data on these trends, especially in routine German care, remain scarce and outdated (Timäus et al., 2021; Loh et al., 2013; Besse et al., 2026).

Beyond overall utilization trends, the indication-specific use of ECT is of particular clinical importance. Clinical guidelines consistently emphasize ECT as a treatment option for therapy-resistant depression. Importantly, it is also considered as an early or even first-line treatment option for psychotic depression, given its superior efficacy compared with pharmacological strategies and other neuromodulatory approaches (Leadholm et al., 2013). Nevertheless, findings from the U.S. suggest that ECT may be underutilized in this population (Case et al., 2013; Kellner et al., 2020). For Germany, recent data on the proportion of patients with psychotic depression receiving ECT are lacking, limiting the ability to assess adherence to guideline recommendations and the adequacy of care.

In addition, substantial regional variation in the availability and use of ECT has been reported internationally, often reflecting differences in infrastructure, expertise, and local clinical practice (Kellner et al., 2020; Barruel et al., 2025; Kaster et al., 2026). Such disparities may result in unequal access to evidence-based treatments, as demonstrated in a recent preprint (Opel et al., 2026). In Germany, however, a comprehensive analysis of regional differences across the different states has not yet been conducted.

Against this background, a detailed and contemporary assessment of neuromodulatory treatment practices in Germany is warranted. The present study therefore pursues three objectives: first, to analyze the development of ECT utilization over the past four years in relation to rTMS; second, to determine the proportion of patients with psychotic depression treated with ECT; and third, to examine regional differences in the use of ECT across all sixteen German states.

## 2 Methods

We conducted a retrospective analysis of nationwide anonymized patient data from German hospitals, reported to the National Institute for the Hospital Remuneration System (InEK GmbH) (Nutzungsumfang, n.d.). Data can be accessed on the InEK DataBrowser (https://datenbrowser.inek.org). The Ethics Committee of the University Hospital Frankfurt has previously waived the requirement for ethical approval and informed consent (Chair: Prof. Dr. Harder, Ref: 2022-766).

Our analyses were conducted within the framework of the prospective payment system for psychiatry and psychosomatics called PEPP. After selecting the desired year and the correct billing system, users access a query interface which provides multiple fields to refine the analysis (such as diagnosis, age and procedure). This enables targeted searches for case numbers. Cases are registered after remission from the hospital. The analyses are based on all data submitted by hospitals within the respective reporting period (Nutzungsumfang, n.d.).

Data was available for the years 2020 to 2025. Primary and secondary diagnosis are coded according to the ICD-10-GM version and procedures according to the German “Operations-und Prozedurenschlüssel OPS” version of the respective year. We concentrated on primary diagnosis for our search. Procedures included repetitive transcranial magnetic stimulation baseline session (OPS code: 8-632.0) and electroconvulsive therapy baseline session (OPS code: 8-630.2). We selected age older than 18 years with no upper limit.

At the beginning, we looked for the total number of ECT and rTMS baseline sessions - meaning the first treatment of a session regardless of the number of treatments during the whole session – a proxy for treated cases. We included the following diagnosis: F31.4 Bipolar disorder, currently severe depressive episode without psychotic symptoms, F31.5 Bipolar disorder, currently severe depressive episode with psychotic symptoms, F31.6 Bipolar disorder, currently mixed episode, F31.0 Single mild depressive episode, F32.1 Single moderate depressive episode, F32.2 Single severe depressive episode without psychotic symptoms, F32.3 Single severe depressive episode with psychotic symptoms, F33.0 Recurrent depressive disorder, currently mild episode, F33.1 Recurrent depressive disorder, currently moderate episode F33.2 Recurrent depressive disorder, currently severe episode without psychotic symptoms, F33.3 Recurrent depressive disorder, currently severe episode with psychotic symptoms and other (F3X.8) as well as not otherwise classified (F3X.9) forms of those disorders. Further search concerning the rate of ECT employment in unipolar recurrent severe depression with psychotic depression (ICD-10-code: F33.3) was carried out. Single severe psychotic depressive episodes played only a marginal role in our dataset and were therefore not analyzed further (not shown). In 2025, for example, only 0.03% (195 of 6465 cases) of all single severe psychotic depressive episodes recorded in Germany involved an ECT baseline session. Afterwards, we examined ECT case numbers regarding specific diagnosis in depressive disorders in 2025. We limited our search strategy to ICD-codes F31.4, F31.5, F32.2, F32.3, F33.2 and F33.3. Lastly, we conducted a regional availability analysis. Therefore, at first, ECT case numbers in 2025 in the 16 German states were enquired for the following diagnosis: F31.4, F31.5, F31.6, F31.8, F31.9, F32.0, F32.1, F32.2, F32.3, F32.8, F32.9, F33.0, F33.1, F33.2, F33.3, F33.8, F33.9. Filtering by state restricts cases to those with discharge locations in the selected state. Secondly, we compared the case numbers to the states’ population (Population Germany, 2025) to report on state-level differences in ECT use. The results were visualized by a heatmap which was created with the help of ChatGPT. Graphics were created and calculation was done with IBM SPSS Statistics (Version 31.0.0.0).

In principle, the InEK DataBrowser allows for the selection of small data subsets; however, for data protection reasons, results with counts fewer than four are not displayed. Thus, to avoid misinterpretation, we chose not to discuss data from the city-states, as hospitals located outside their administrative boundaries may be responsible for treating patients residing within them. The resulting small case numbers could also be suppressed by the InEK DataBrowser to protect against re-identification. Furthermore, to prevent identification of individual patients despite anonymization, the dataset was not only minimized to the variables necessary for analysis but also partially modified. Instead of identifying individual hospitals, cases were assigned to categories based on hospital bed size and ownership. Exact patient age was replaced by age groups, and precise timestamps (date and time) were reduced to date-only information (Nutzungsumfang, n.d.).

Sentence structure was partly revised by generative AI.

## 3 Results

### 3.1 Temporal trends

To evaluate temporal trends in neuromodulatory treatments, we first assessed the total annual number of ECT and rTMS in Germany between 2020 and 2025 among adult patients (>18 years) with any depressive disorders (for more detailed information on the ICD10 codes see Methods section). As shown in Fig. 1, both interventions exhibited a rise in utilization across the study period. The average annual growth rate for rTMS was 35.39% (minimum: 15.6% in 2022; maximum: 62.1% in 2021), markedly exceeding that of ECT (4.88% (minimum: −0.2% in 2021; maximum: 12.8% in 2023)).

**Fig. 1:**
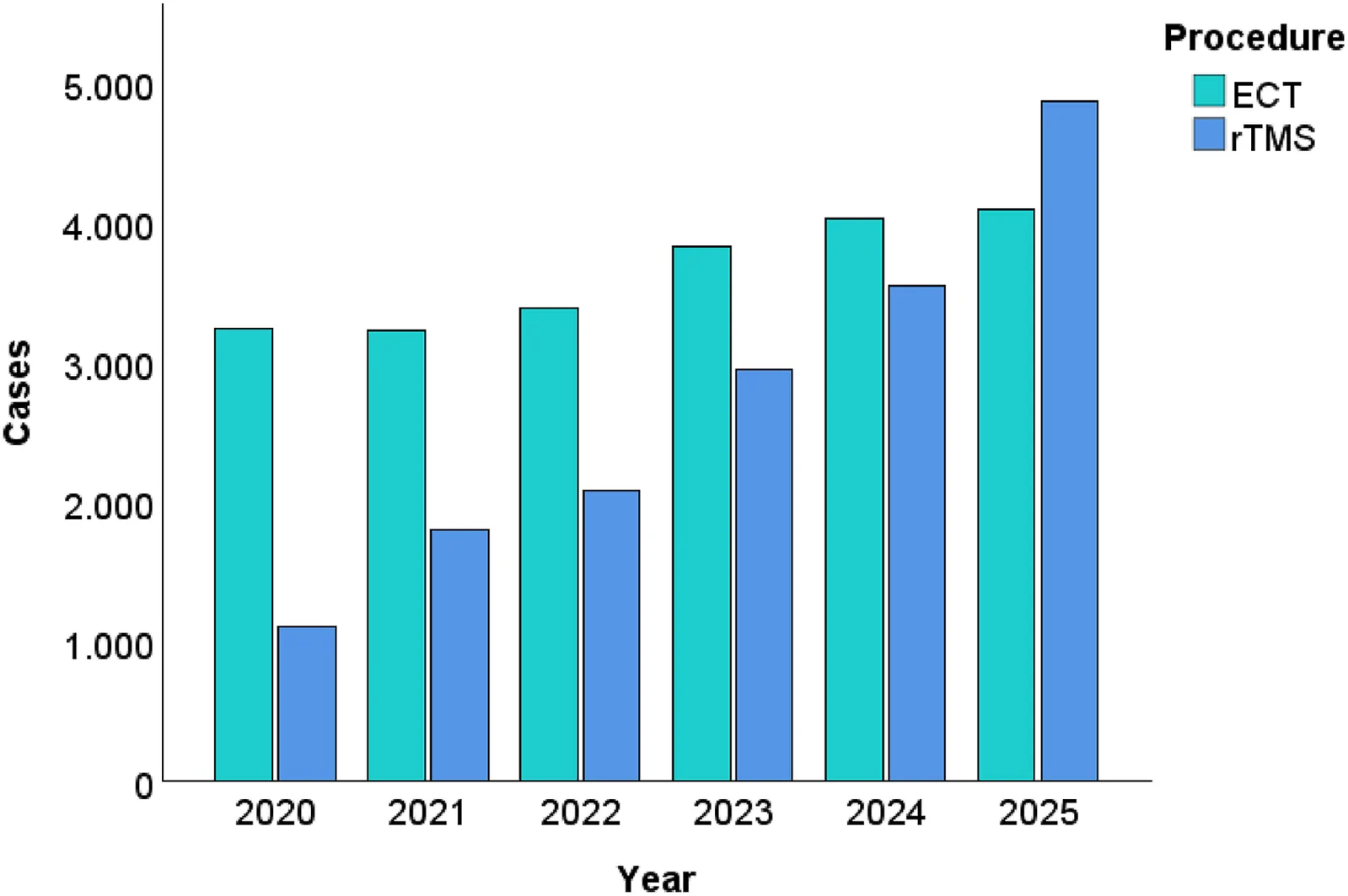
Temporal trends of ECT and rTMS: Number of ECT and rTMS baseline sessions in depressed patients (age > 18 years) with at least one ECT session per year in Germany from 2020 to 2025.

Next, we analyzed the proportion of patients suffering from recurrent depressive disorder, current severe episode with psychotic symptoms (ICD-10-code: F33.3) on receiving ECT relative to all registered cases of this disorder (Fig. 2). Across the five-year period, we found a constant increase with an average growth rate of 6.0% per year (minimum: 0,7% in 2025; maximum: 12.0% in 2023). In 2020, ECT was only performed in 498 cases of recurrent psychotic depression - compared to 724 cases in 2025 accounting for 4.1% and 5.5% of all cases respectively.

**Fig. 2:**
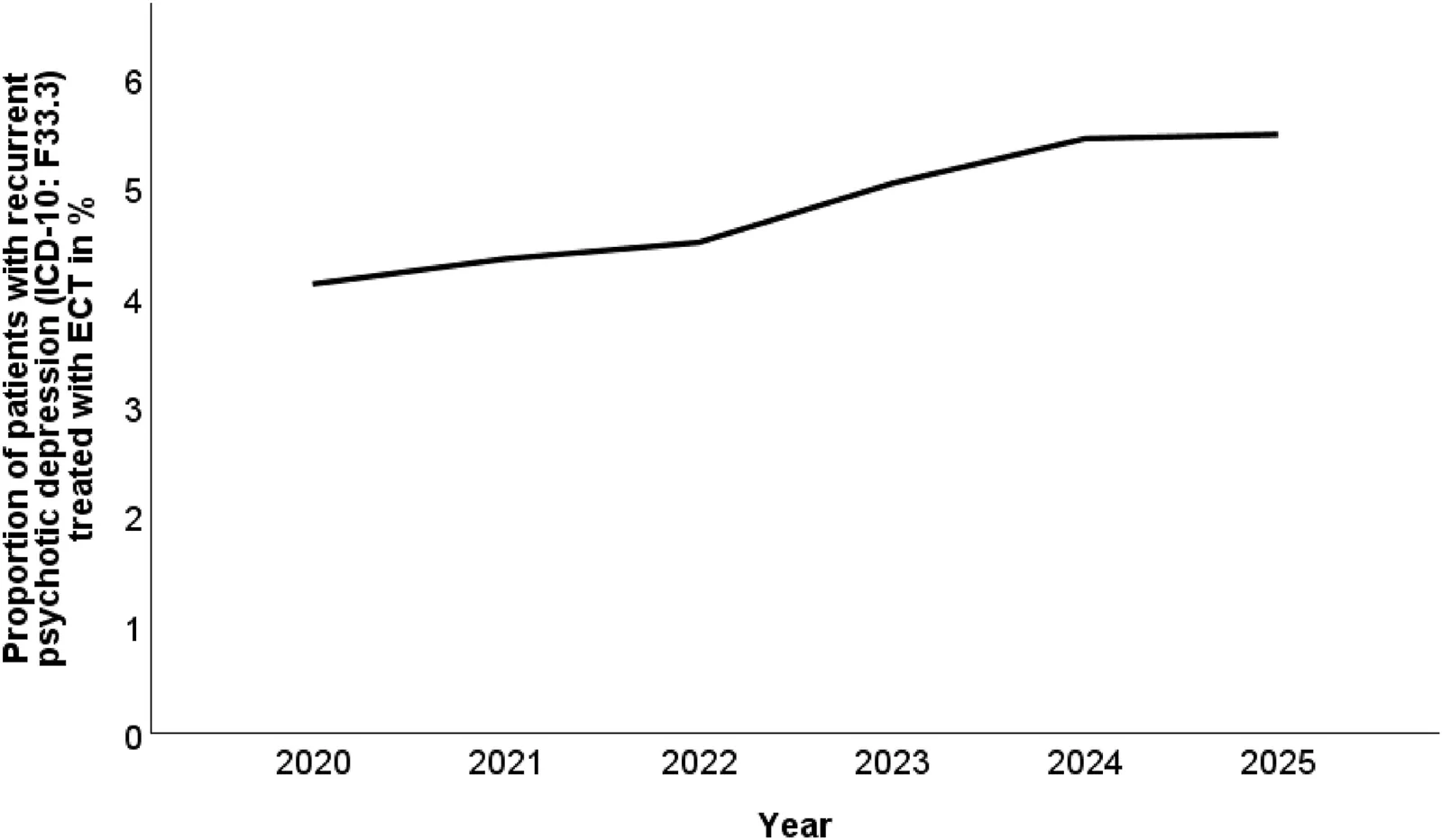
Percentage of recurrent severe psychotic depression cases (ICD-10-code: F33.3) treated with ECT in German adult patients from January 2020 to December 2025.

Further analyses focused on ECT use within the broader patient population diagnosed with severe depressive episodes, defined by ICD-10-codes F31.4, F31.5, F32.2, F32.3, F33.2, and F33.3 (Fig. 3). The overall increase in ECT cases was primarily driven by patients with recurrent severe non-psychotic depression (ICD-10-code F33.2; + 545 baseline sessions (+32.97%) comparing 2020 with 2025), followed by those with recurrent severe psychotic depression (ICD-10-code F33.3; + 226 baseline sessions (+45.38%) comparing 2020 with 2025) and single severe depressive episodes (ICD-10-code F32.2; + 74 baseline sessions (+43,53%) comparing 2020 with 2025) as well as bipolar severe depression without psychotic symptoms (ICD-10-code F31.4; + 72 baseline sessions (+21,11%) comparing 2020 with 2025). The total number of registered cases during the period regarded grew for every diagnosis. Cases of recurrent severe non-psychotic depression increased by 38.56% (ICD-10-code F33.2; 111.818 cases in 2020 compared to 154.938 cases in 2025), by 8.97% in recurrent severe psychotic depression (ICD-10-code F33.3; 12.114 cases in 2020 compared to 13.201 cases in 2025), by 15.8% in single severe non-psychotic depression (ICD-10-code F32.2; 55.759 cases in 2020 compared to 64.569 cases in 2025) and by 20.46% in bipolar severe depression without psychotic symptoms (ICD-10-code F31.4; 7.978 cases in 2020 compared to 9.610 cases in 2025).

**Fig. 3:**
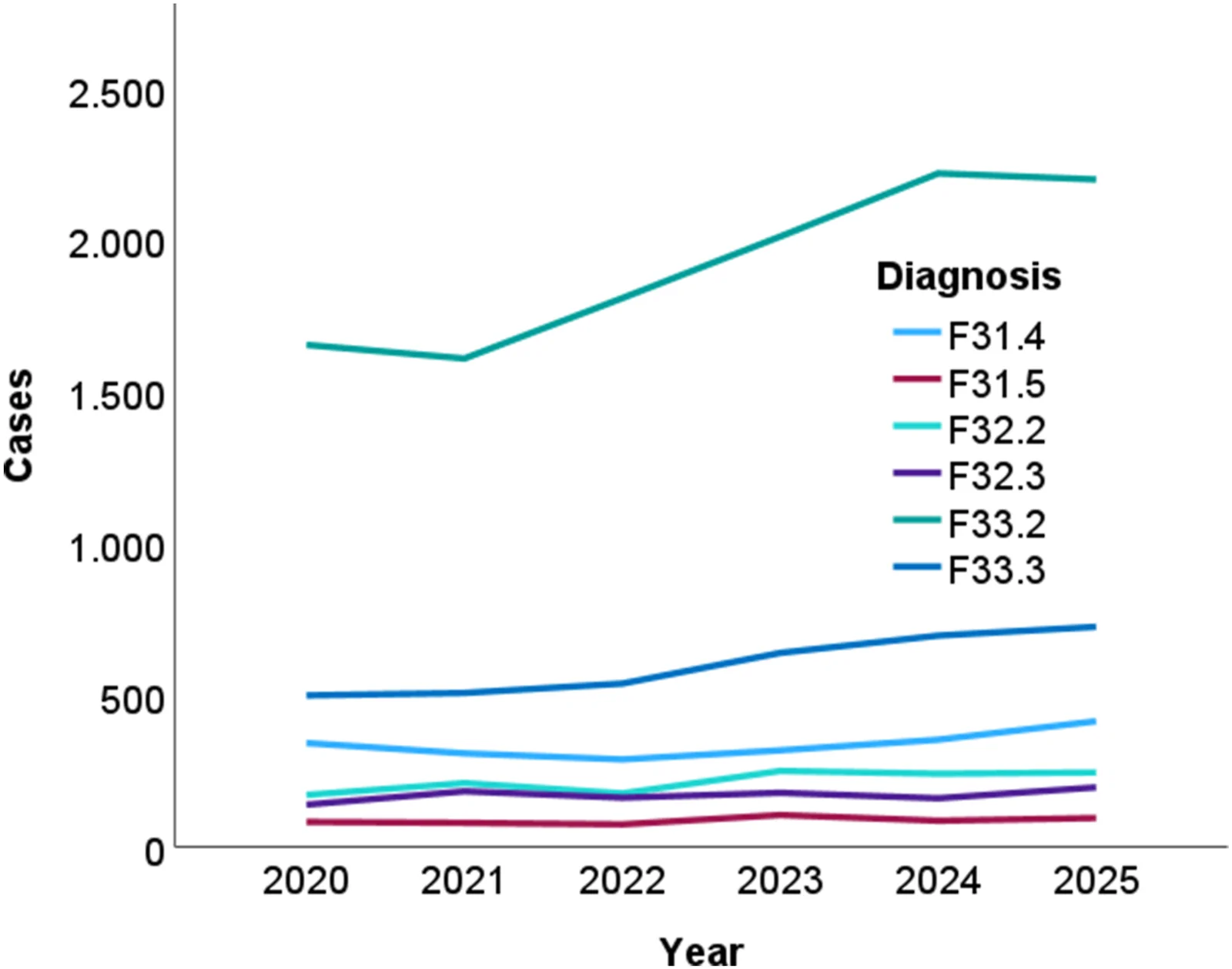
ECT according to diagnosis: ECT cases in an adult patient population in Germany from January 2020 to December 2025 stratified by diagnosis: F31.4 Bipolar disorder, currently severe depressive episode without psychotic symptoms, F31.5 Bipolar disorder, currently severe depressive episode with psychotic symptoms, F32.2 Single severe depressive episode without psychotic symptoms, F32.3 Single severe depressive episode with psychotic symptoms, F33.2 Recurrent depressive disorder, currently severe episode without psychotic symptoms, F33.3 Recurrent depressive disorder, currently severe episode with psychotic symptoms.

### 3.2 Regional availability analysis

Finally, a heatmap was generated to depict the regional distribution of ECT cases in 2025 (restricted to adult patients with depressive episodes (for more detailed information on the ICD10 codes see Methods section)) across Germany's sixteen states (Fig. 4) in relation to their overall population at the end of that year. The analysis revealed almost a threefold difference in ECT availability - ranking from 2.45 to 7.26 cases per one hundred thousand inhabitants in different states in Germany (city states excluded from the analysis).

**Fig. 4:**
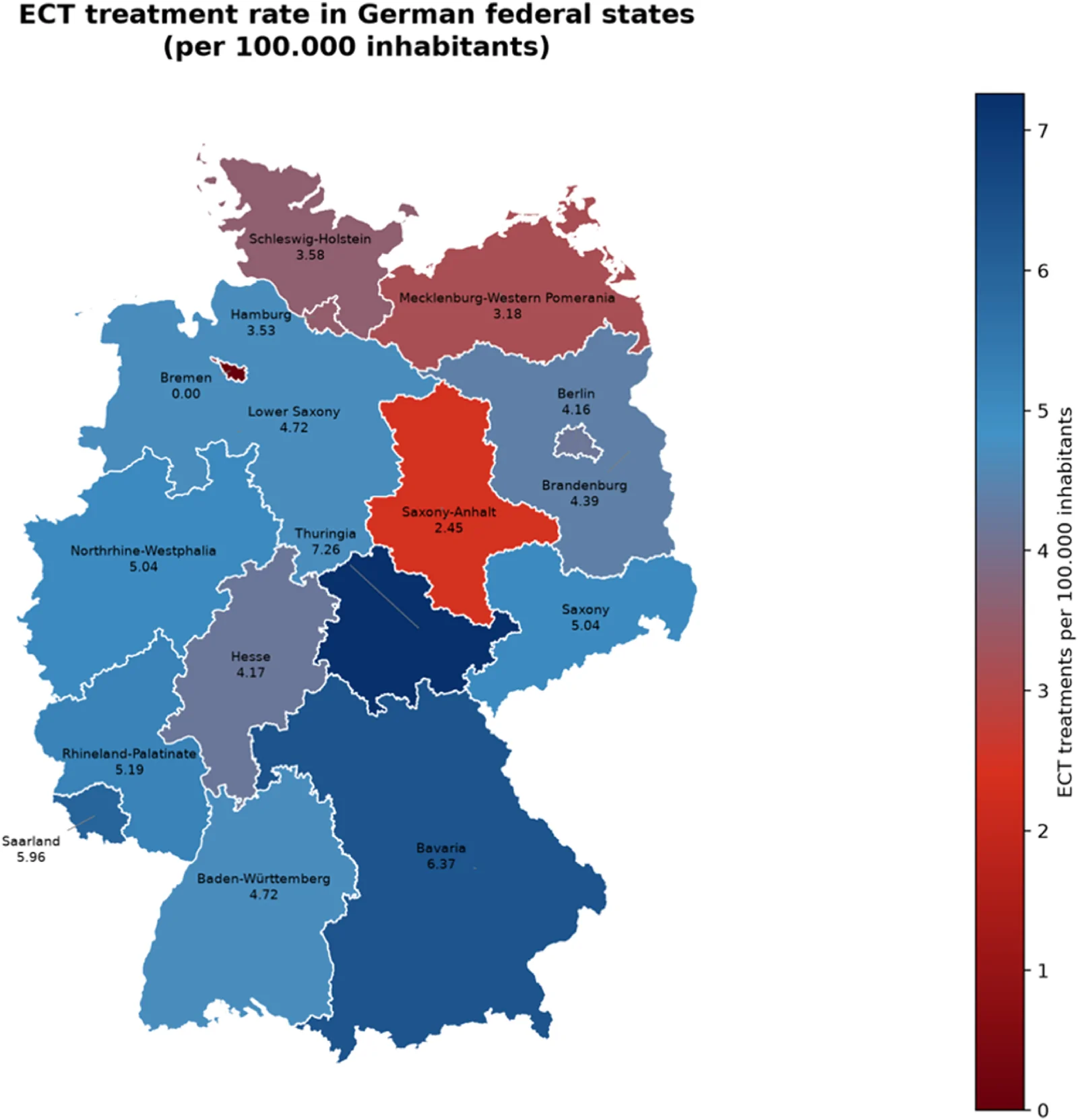
ECT Heatmap: ECT cases in depressive adult patients in relation to 100.000 inhabitants in German states from January to December 2025.

## 4 Discussion

We demonstrated that, based on nationwide hospital remuneration data, utilization of both electroconvulsive therapy (ECT) and transcranial magnetic stimulation (TMS) has increased in recent years, whereas the proportion of patients with psychotic depression treated with ECT has remained low at approximately 5.5%, and considerable inter-state variation in ECT use persisting across Germany.

Repetitive transcranial magnetic stimulation (rTMS) implementation in clinical practice has markedly expanded over the past five years, likely reflecting the revised guideline for unipolar depression (Nationale Versorgungs Leitlinie Unipolare Depression, n.d.), which includes rTMS as a treatment option for treatment-resistant unipolar depression, as well as the availability of specific reimbursement for rTMS procedures in Germany since 2021 (Schecklmann et al., 2025). Notably, by 2025, rTMS-treated patients outnumbered those receiving ECT, with ECT numbers showing a slower growth trajectory despite its long-standing evidence for high efficacy in severe, treatment-resistant and psychotic depression. Importantly, on an aggregate level, the increase in rTMS does not appear to have coincided with a decrease in ECT. ECT growth was primarily driven by greater uptake in recurrent and single episode severe non-psychotic depression as well as from recurrent severe psychotic depression, while utilization for other diagnostic groups remained broadly stable. RTMS growth was amplified by a sharp rise in non-psychotic unipolar depression (not shown). This is in line with current clinical reality that suggests that ECT and rTMS target partially different patient populations with ECT still being the gold-standard for the most severe cases, especially for catatonic and psychotic depression (Zilles-Wegner et al., 2024; Espinoza et al., 2025).

In contrast to these German trends, ECT utilization worldwide is heterogenous. Overall use concentrates in high-income settings(Leiknes et al., 2012) but higher use per admission is registered in some economically emerging countries as Nepal (Ahikari et al., 2008) or Malawi (Selis et al., 2008). International data cited in the literature showed very wide variation in national rates, from 0.11 per 10,000 inhabitants in Poland to 5.10 in the USA (Leiknes et al., 2012). Scandinavian register data have revealed a use of ECT in inpatient care ranging from 1.7 to 5.3% (Leiknes et al., 2012). Lately, a decline was registered independent of the indication in the USA (Lesage et al., 2016). In an overview, 62% of patients were treated because of a major depressive disorder (in 25% additionally suffering from psychotic symptoms) (Rohde et al., 2024). In western countries, recipients tended to be older women with depression whereas schizophrenia predominated in Asia in the available data (Leiknes et al., 2012). No comparable global data exist for the utilization of rTMS.

Beyond national trends, our state-level analysis aimed to characterize regional care provision by mapping baseline ECT sessions per one hundred thousand inhabitants across the 16 German states. This analysis revealed pronounced regional disparities, with rates ranging from 2.45 to 7.26 cases per one hundred thousand inhabitants (city states excluded for methodological reasons), corresponding to almost a threefold difference in ECT utilization within the same healthcare system. Intriguingly, the discrepancies in regional disparities in Germany only partially align with common patterns of healthcare access and utilization: states with typically weaker healthcare infrastructure - as described for neurological acute care in Rhineland-Palatinate and Thuringia for instance - show the highest per-capita-rates (Masanneck et al., 2026). The findings suggest that access to ECT in Germany is strongly influenced by regional service structures and local implementation practices, raising important questions about equity of care and adherence to guideline-based treatment pathways for severe and psychotic depression.

This is especially alarming given guideline recommendations that emphasize ECT as a key treatment option for psychotic depression, with five out of nine guidelines prioritizing ECT as a treatment for psychotic depression (Leadholm et al., 2013), ECT was administered in only about 5.5% of such cases in Germany, suggesting a startling under-use of this method in a population with only few other treatment alternatives. Potential explanations for this underutilization are multifactorial and may include the limited number of ECT-capable centers, consequent lack of clinician experience and confidence, and persistent societal and professional reservations, partly fueled by stigmatizing or misleading public discourse (Zilles-Wegner et al., 2026; Lauber et al., 2005; Espinoza et al., 2025; Paheenthararajah et al., 2015). We therefore strongly support the ambition to further promote ECT implementation in the training of psychiatrists in Germany.

A possible limitation of our analysis is that only data from baseline sessions as a proxy for the total number of ECT sessions was assessed, leaving the actual number of completed sessions veiled. Furthermore, because InEK DataBrowser sets high standards regarding data security, it does not capture unique patients or detailed clinical information. Due to those regulations, case numbers smaller than four are not displayed in the search, which could influence the analysis. Especially in smaller states, like Bremen, where no ECT baseline sessions have been registered for unipolar depressive episodes of any given severity, data could be incomplete. In addition, coding practices, and regional differences in service availability may bias estimates of ECT and rTMS utilization.

Lastly, the analyses were conducted using data from 2020 to 2025. During this period, the Coronavirus Disease 2019 (COVID-19) pandemic affected the availability of ECT, as described by Karl et al. (2022). Thus, the results of the present study may have been influenced or confounded by the exceptional circumstances of the pandemic; however, the InEK DataBrowser only provided access to data from 2020 onward, so an earlier baseline could not be obtained.

To further contextualize the development of ECT and rTMS utilization, we conducted an additional literature search for comparable pre-pandemic data. Given the differences in study periods, populations, and methodologies, these comparisons can only be interpreted as approximate reference points rather than as evidence of a direct temporal trend (Loh et al., 2013; Nordanskog et al., 2015). Additionally, we found no comparable pre-pandemic data on rTMS utilization within the relevant time frame.

As this is the first study to use the InEK DataBrowser to investigate the use of ECT and rTMS in Germany, direct comparisons with other epidemiological findings on ECT use should be interpreted with caution. Consequently, we were unable to assess the effect of the pandemic in a scientifically reliable manner.

## 5 Conclusion

ECT and rTMS are effective and increasingly utilized neuromodulatory treatments for depressive disorders, as reflected by the substantial growth in cases over the past years. Growing numbers of both procedures underscore their clinical relevance but also reveal considerable regional disparities in access, indicating a need to expand the use of ECT in several German states. In light of the robust evidence base, ECT should be used more consistently as a treatment option for severe depression, particularly recurrent psychotic depression, in accordance with current guideline recommendations. The data analyzed, for instance, showed an underrepresentation of ECT in psychotic depression. Increasing the number of ECT-capable centers could facilitate structured training of young psychiatrists, promote greater familiarity with the procedure, and help counter persistent stigma and misconceptions. Momentarily, availability of ECT remains restricted due to a lack of centers, making access to ECT treatment dependent on the geographical area. Parallel efforts in public education are essential to improve acceptance of neuromodulatory treatments and to ensure equitable, guideline-concordant care for patients with severe and treatment-resistant mood and psychotic disorders.

## Ethics declaration

The authors have NOT obtained informed consent from participants or their legal representatives. Explanation: German hospitals are required by law to report data of all patients in an anonymized form to InEK (§ 21 KHEntgG). Datasets are anonymized and are open for scientific research according to German law. Due to the full anonymization of the data, informed consent was not required. Therefore, the Ethics Committee of the University Hospital Frankfurt waived the requirement for informed consent.

This study was performed in compliance with relevant laws, regulatory frameworks and guidelines where the research took place. This study was approved by the University Hospital Frankfurt/Main. (Approval No. 2022-766)

## Declaration of generative AI and AI-assisted technologies in the manuscript preparation process

During the preparation of this work the authors used ChatGPT in order to generate a heatmap of the utilization of ECT across German states. After using this tool, the authors reviewed and edited the content as needed and take full responsibility for the content of the published article.

## Declaration of competing interest

The authors declare the following financial interests/personal relationships which may be considered as potential competing interests: Jonathan Repple received speaker's honoraria from Janssen, Hexal, Neuraxpharm and Novartis. Marius Gruber received remuneration from Janssen for consultancy services. Andreas Reif has received honoraria for lectures and/or advisory boards from Janssen/Johnson & Johnson, Boehringer Ingelheim, Compass, GH Research, SAGE/Biogen, LivaNova, Medice, Shire/Takeda, Newron, MSD, AbbVie, Schwabe and Polpharma. Also, he has received research grants from Medice and Janssen. Christine Reif-Leonhard received honoraria from J&J, Medice, Abbvie and LivaNova. If there are other authors, they declare that they have no known competing financial interests or personal relationships that could have appeared to influence the work reported in this paper.
